# Targeting Cancer Hallmarks with *Cirsium japonicum*: Molecular Mechanisms and Therapeutic Potential

**DOI:** 10.3390/ijms27187996

**Published:** 2026-09-08

**Authors:** Kyung-Hee Kim, Tae-Kyung Yeo, So-Jung Park, Hwa-Seung Yoo, Byong Chul Yoo

**Affiliations:** 1 Department of Applied Chemistry, School of Science and Technology, Kookmin University, Seoul 02707, Republic of Korea; kyungheekim@kookmin.ac.kr; 2 Antibody Research Institute, Kookmin University, Seoul 02707, Republic of Korea; 3 Oculi Korean Medicine Hospital, Seoul 05655, Republic of Korea; oculi2022@naver.com; 4 Department of Korean Internal Medicine, School of Korean Medicine, Pusan National University, Yangsan 50612, Republic of Korea; vivies@daum.net; 5 East West Cancer Center, Daejeon Korean Medicine Hospital, Daejeon University, Daejeon 34520, Republic of Korea; 6 Institute of Biotechnology and Bioengineering, Sungkyunkwan University, Suwon 16419, Republic of Korea

**Keywords:** *Cirsium japonicum*, cancer hallmarks, natural products, flavonoids, pectolinarigenin, regulated cell death, tumor microenvironment, therapeutic resistance

## Abstract

*Cirsium japonicum* is a medicinal plant traditionally used in East Asian medicine and contains multiple bioactive constituents with potential anticancer properties. This review critically evaluates the anticancer effects of *C. japonicum* and its associated constituents within the Hallmarks of Cancer framework. Evidence was classified as direct plant-specific evidence, constituent-based evidence, or contextual mechanistic evidence to distinguish findings obtained using botanical preparations from those generated using purified compounds derived from other sources. Direct studies of *C. japonicum* extracts, flavonoid fractions, phenylpropanoid glycosides, and *C. japonicum* var. *maackii*-mediated gold nanoparticles demonstrate antitumor, cytotoxic, antiangiogenic, immunomodulatory, and ferroptosis-associated activities, although target-level validation remains limited. Among its associated constituents, pectolinarigenin has the most developed mechanistic evidence, involving RRM2–CDK1, TOP2A, STAT3, and PI3K/AKT/mTOR signaling. Apigenin, luteolin, acacetin, linarin, cirsimaritin, and pectolinarin additionally influence regulated cell death, proliferative signaling, angiogenesis, metastasis, immune regulation, metabolic vulnerability, senescence, and therapy response. However, much of this evidence relies on pathway-associated changes rather than direct target engagement or genetic validation. Furthermore, the pharmacokinetics, systemic exposure, botanical standardization, tumor selectivity, and clinical relevance of these compounds remain insufficiently characterized. Future studies should prioritize standardized preparations, quantitative exposure analysis, functional target validation, clinically relevant models, and carefully designed combination strategies. Collectively, *C. japonicum* provides a promising multi-hallmark framework for anticancer investigation, but substantial translational evidence is still required before its therapeutic potential can be established.

## 1. Introduction

Cancer progression is driven by interconnected biological capabilities that allow malignant cells to sustain proliferation, resist regulated cell death, reprogram metabolism, remodel the tumor microenvironment, invade distant tissues, and evade immune destruction. The Hallmarks of Cancer framework provides a practical structure for integrating these processes and has progressively expanded to encompass metabolic reprogramming, immune evasion, phenotypic plasticity, non-mutational epigenetic reprogramming, senescent cells, and tumor–microenvironment interactions [1,2,3]. Tumor progression is therefore not determined solely by cancer-cell-intrinsic alterations but also by reciprocal interactions with stromal, vascular, and immune components [4,5]. Because these biological processes are functionally interconnected, inhibition of a single pathway may be offset by signaling redundancy, metabolic plasticity, or microenvironmental support [6,7,8]. These limitations provide a rationale for investigating chemically diverse agents capable of modulating multiple interconnected processes rather than a single isolated target.

In this context, natural products remain important sources of pharmacologically active compounds and chemical scaffolds for anticancer drug development [9,10]. Their structural diversity and capacity to influence multiple signaling networks may be advantageous in heterogeneous tumors, in which parallel pathways frequently sustain malignant phenotypes. However, multi-target activity also creates a major interpretive challenge. Changes in kinase phosphorylation, apoptosis-related proteins, oxidative stress, or cytokine expression are often presented as molecular mechanisms even when direct target engagement or pathway dependency has not been demonstrated. This problem is particularly relevant to medicinal plants, for which the biological activities of widely distributed phytochemicals may be attributed to the plant itself without evidence that a botanical preparation contains or delivers pharmacologically effective concentrations.

*Cirsium japonicum* Fisch. ex DC. is a medicinal plant of the Asteraceae family traditionally used in East Asian medicine for hemorrhagic, inflammatory, and hepatic disorders. Phytochemical analyses of *C. japonicum* have identified pectolinarin, linarin, luteolin, and related flavonoids [11], whereas apigenin, luteolin, apigenin 7-*O*-glucuronide, and luteolin 5-*O*-glucoside have been isolated from *C. japonicum* var. *maackii* [12]. Structurally distinctive phenylpropanoid glycosides have also been isolated directly from *C. japonicum* [13]. Pectolinarigenin, the aglycone of pectolinarin, is included as an associated constituent, although most of its anticancer evidence was generated using purified material rather than standardized *C. japonicum* preparations. This phytochemical evidence supports their inclusion in the present review; however, the anticancer studies of these compounds were generally performed using commercially sourced materials or compounds obtained from other botanical sources and are therefore classified as constituent-based rather than direct plant-specific evidence. Representative preclinical studies of *C. japonicum* and its associated constituents, together with their experimental models, principal findings, mechanistic validation, and evidence categories, are summarized in Table 1.

One study compared a total flavone preparation from *C. japonicum* (FLCJ) with pectolinarin and 5,7-dihydroxy-6,4′-dimethoxyflavone and associated their inhibition of S180 and H22 tumor growth with enhanced cellular and humoral immune responses [14]. A separate study isolated and quantified the two flavones in methanol, ethanol, and aqueous extracts and evaluated their effects on tumor growth and survival in S180- and H22-bearing mice [15]. Other direct plant-specific studies examined isolated phenylpropanoid glycosides, an ethanol extract and its associated constituent cirsimaritin, and plant-mediated gold nanoparticles [13,16,17,60]. By contrast, most target-oriented evidence has been generated using isolated or purified constituents that were either commercially obtained or derived from botanical sources other than *C. japonicum*, particularly pectolinarigenin, pectolinarin, acacetin, apigenin, luteolin, linarin, and cirsimaritin [18,19,20,21,22,23,24,25,26,27,28,29,30,32,33,34,35,36,37,38,39,40,41,42,43,44,45,46,47,48,49,50,51,52,53,54,55,56,57,58,59]. Because these studies differ substantially in plant specificity and experimental strength, their principal findings are summarized in Table 1 and critically evaluated in the relevant sections below. Some incorporate target engagement assays, genetic rescue, pharmacological inhibition, orthotopic tumors, metastasis models, immunocompetent hosts, or combination treatments, whereas others rely mainly on cytotoxicity assays and changes in protein expression or phosphorylation. The strengths and limitations of the current evidence are critically evaluated throughout this review.

A further distinction is essential throughout this review. Evidence obtained using *C. japonicum* extracts, fractions, or compounds explicitly isolated from the plant is classified as direct plant-specific evidence. Studies using commercially sourced compounds or compounds isolated from other botanical species are classified as constituent-based evidence. General studies defining the biological roles of cancer-associated pathways provide contextual mechanistic evidence. Constituent-based studies can identify plausible therapeutic mechanisms, but they do not establish that conventional *C. japonicum* preparations achieve equivalent exposure or reproduce the same biological effects.

This review therefore critically evaluates the anticancer pharmacology of *C. japonicum* and its associated constituents through the Hallmarks of Cancer framework. Rather than describing every experimental result individually, the evidence is integrated across proliferative signaling, regulated cell death, inflammation, invasion and metastasis, angiogenesis, immune evasion, metabolic vulnerability, senescence, phenotypic plasticity, and therapy resistance. Particular emphasis is placed on distinguishing validated molecular dependencies from correlative signaling changes and on identifying the pharmacokinetic, methodological, and translational barriers that must be addressed before *C. japonicum*-derived interventions can advance toward mechanism-based cancer therapy.

## 2. Bioactive Constituents and Evidence Categories

The anticancer literature associated with *C*. *japonicum* can be divided into three evidence categories: direct studies using plant extracts, fractions, or compounds isolated from *C. japonicum*; constituent-based studies using commercially sourced compounds or compounds isolated from other botanical species; and contextual studies that clarify general cancer mechanisms without directly testing the plant. These categories are not equivalent and should be interpreted separately. In particular, computational predictions, cytotoxicity assays, pathway-associated protein changes, and experimentally validated molecular dependencies represent different levels of evidence. These evidence categories provide the conceptual framework for the following sections.

The taxonomic treatment of Korean thistle varies among botanical authorities. World Flora Online recognizes *Cirsium japonicum* var. *maackii* (Maxim.) Matsum. as an accepted infraspecific taxon of *C. japonicum*, with *Cirsium maackii* Maxim. treated as a synonym [61]. By contrast, some regional floristic treatments recognize *C. maackii* as a separate species [62]. In the present review, the nomenclature reported in each original study has been retained, and evidence obtained using *C. japonicum* var. *maackii* is explicitly identified as such. These studies are considered within the broader *C. japonicum* complex for comparative purposes but are not assumed to be botanically or pharmacologically interchangeable with studies using *C. japonicum* sensu stricto.

The two taxa show overlapping but not necessarily identical phytochemical profiles. Analyses of *C. japonicum* have identified pectolinarin, linarin, luteolin, and related flavonoids [11], whereas apigenin, luteolin, and their glycosylated derivatives have been isolated from *C. japonicum* var. *maackii* [12]. This overlap provides a rationale for comparative discussion within the same review; however, differences in constituent abundance and the absence of systematic side-by-side profiling under matched conditions preclude assuming chemical equivalence. Accordingly, findings derived from *C. japonicum* var. *maackii* are distinguished throughout the text and tables from those obtained using *C. japonicum* sensu stricto.

### 2.1. Direct Plant-Specific Evidence

Direct anticancer evidence for *C. japonicum* remains limited but includes both cellular and animal studies. Together, these studies provide direct in vivo evidence for the antitumor activity of *C. japonicum* flavone preparations and isolated constituents but do not identify direct molecular targets or establish tumor selectivity, systemic exposure, or the relative contribution of individual compounds [14,15].

Phenylpropanoid glycosides isolated from the aerial parts of *C. japonicum* exhibited low-micromolar cytotoxicity against MCF-7 breast cancer, U87 glioma, HCT116 colorectal cancer, and A549 lung cancer cells [13]. Subsequent total synthesis of one of the newly identified cytotoxic glycosides confirmed its chemical structure and provided a route for future structure–activity and mechanistic studies [60]. Nevertheless, the original biological investigation primarily assessed cytotoxicity; the responsible death mechanisms, direct molecular targets, in vivo efficacy, and normal-cell selectivity remain unknown.

An ethanol extract of *C. japonicum* var. *maackii* and its major constituent cirsimaritin reduced endothelial cell viability and tube formation and suppressed VEGF-, AKT-, and ERK-associated signaling [16]. These findings support an antiangiogenic phenotype but do not demonstrate inhibition of spontaneous metastasis or direct engagement of VEGF-pathway components. The same botanical variety has also yielded cirsimaritin with experimentally demonstrated anti-inflammatory activity, providing contextual support for its capacity to regulate inflammation-associated signaling, although that study did not directly evaluate cancer [63].

More recently, gold nanoparticles synthesized using *C. japonicum* var. *maackii* extract induced ROS and Fe^2+^ accumulation, lipid peroxidation, GPX4-associated antioxidant disruption, and ferroptosis-like death in AGS gastric cancer cells and inhibited xenograft growth [17]. Because the active material is a hybrid nanostructure, these effects cannot be attributed exclusively to the botanical extract. Nanoparticle size, uptake, surface chemistry, and the gold core may all contribute to the observed redox and ferroptotic responses.

A network pharmacology analysis has additionally proposed potential anticancer targets and signaling pathways for *C. japonicum* var. *maackii* flowers [64]. Such findings may assist hypothesis generation but should remain classified as computational evidence unless supported by direct target engagement and functional validation experiments.

Overall, direct studies demonstrate antitumor potential but remain mechanistically underdeveloped. Most target-oriented evidence has instead been generated using purified constituents. The major direct plant-specific and constituent-based studies are summarized in the consolidated Table 1.

### 2.2. Pectolinarigenin and Pectolinarin

Pectolinarigenin is currently the most extensively studied *C. japonicum*-associated constituent. It has been evaluated in nasopharyngeal, lung, breast, liver, brain, and bladder cancer models. Reported effects include G2/M arrest, mitochondrial apoptosis, autophagy regulation, DNA damage, reduced invasion and metastasis, and treatment sensitization [18,19,20,21,22,23]. More recently, pectolinarigenin also exhibited potent anticancer activity in triple-negative breast cancer through G0/G1 cell-cycle arrest, apoptosis, and MAPK signaling inhibition [24].

The strongest mechanistic evidence involves the RRM2–CDK1 axis in glioblastoma. Pectolinarigenin increased autophagic flux and promoted degradation of RRM2 and CDK1, whereas RRM2 overexpression partially reversed cell-cycle arrest and growth inhibition. The inclusion of orthotopic and subcutaneous models further strengthened the preclinical relevance of this mechanism [20].

In bladder urothelial carcinoma, pectolinarigenin induced TOP2A-associated DNA damage, activated p53-related G2/M checkpoint signaling, altered late autophagic flux, and enhanced gemcitabine activity [21]. However, definitive classification as a TOP2A poison requires biochemical demonstration that the compound stabilizes TOP2A–DNA cleavage complexes rather than merely altering TOP2A-associated activity or expression.

Pectolinarigenin has also been associated with inhibition of PI3K/AKT/mTOR and ERK signaling in hepatocellular carcinoma, PTEN–PI3K/AKT-associated malignant phenotypes in non-small-cell lung cancer, and STAT3-associated metastatic signaling in breast cancer [19,22,23]. In the breast cancer model, suppression of pulmonary metastasis was accompanied by reduced MMP-2 and MMP-9, increased TIMP2, and enhanced CD8^+^ T-cell recruitment [19]. These studies support pathway involvement, although direct target engagement was not demonstrated.

Additional gastric cancer xenograft research has associated pectolinarigenin treatment with alterations in tumor-related proteins, extending its in vivo evidence beyond the better-characterized glioblastoma, breast cancer, and bladder cancer models [25]. Nevertheless, expression profiling alone does not establish which altered protein represents the primary pharmacological target.

Pectolinarin, the glycosylated form of pectolinarigenin, has substantially weaker anticancer evidence. Cytotoxicity and structure–activity relationships have been reported in several tumor cell models [26], whereas ER-stress-associated signaling involving activating transcription factor 6 (ATF6), protein kinase RNA-like endoplasmic reticulum kinase (PERK)–eukaryotic translation initiation factor 2 alpha (eIF2α), and inositol-requiring enzyme 1 (IRE1) has been observed in PC12 cells [27]. The latter constitutes contextual mechanistic evidence rather than a conventional anticancer study. Pectolinarin has also inhibited proliferation and induced apoptosis in rheumatoid arthritis fibroblast-like synoviocytes through PI3K/AKT-associated signaling, but this nonmalignant model should not be treated as direct cancer evidence [28].

Differences in glycosylation can substantially influence intestinal hydrolysis, membrane permeability, systemic exposure, and intracellular activity. Pectolinarin and pectolinarigenin should therefore not be treated as pharmacologically interchangeable without comparative pharmacokinetic and efficacy data.

### 2.3. Apigenin and Luteolin

Apigenin and luteolin have been detected in phytochemical analyses of *C. japonicum* var. *maackii*, providing a botanical basis for their inclusion in this review [12]. Nevertheless, both are widely distributed flavonoids, and the anticancer studies discussed below generally used commercially sourced compounds or materials obtained from other botanical species. Their reported anticancer activities are therefore classified as constituent-based mechanistic evidence and should not be interpreted as direct evidence for *C. japonicum* preparations.

Apigenin has been linked to PI3K/AKT/mTOR inhibition, death receptor sensitization, protective autophagy, Nrf2-associated drug resistance, HIF-1α/VEGF regulation, and GLUT1-associated radiosensitivity [32,33,36,39,40]. In hepatocellular carcinoma, ATG5 silencing or pharmacological autophagy inhibition enhanced apigenin-induced apoptosis, demonstrating that autophagy functioned as a cytoprotective response [32]. In doxorubicin-resistant HCC, apigenin reduced PI3K/AKT/Nrf2-associated antioxidant defense and miR-520b–ATG7-mediated protective autophagy, thereby restoring chemosensitivity [33,34,35]. Apigenin has also sensitized malignant cells to TRAIL, cisplatin, and 5-fluorouracil through death-receptor- and mitochondrial-apoptosis-associated mechanisms [36,37].

Luteolin has shown broader hallmark coverage. DR5 silencing and soluble receptor blockade demonstrated a functional requirement for death receptor signaling in luteolin-induced apoptosis [41]. Luteolin has also induced ER-stress-associated apoptosis and noncanonical autophagy in lung cancer, promoted p53-dependent growth suppression in colorectal cancer, and enhanced radiation-induced apoptosis through a p38/ROS/caspase cascade [45,46,47].

Its broader activities include HO-1-associated ferroptosis in clear-cell renal cell carcinoma, aryl hydrocarbon receptor (AhR)–C-X-C motif chemokine receptor 4 (CXCR4)–matrix metalloproteinase (MMP)-associated inhibition of metastasis, VEGFR2- and integrin β1-associated suppression of angiogenesis, and modification of IL-6/STAT3- and TGF-β1-dependent communication between glioma stem cells and macrophages [42,43,44,48,49]. These findings provide broad mechanistic support but should not be interpreted as evidence that conventional *C. japonicum* preparations reproduce the same effects or achieve equivalent tissue exposure.

### 2.4. Acacetin, Linarin, and Cirsimaritin

Acacetin was detected in rat plasma following oral administration of a *C. japonicum* DC. extract, supporting its phytochemical association with *C. japonicum* sensu stricto [50]. However, the anticancer studies reviewed here did not evaluate acacetin isolated directly from *C. japonicum* and are therefore classified as constituent-based evidence. Acacetin has a smaller but comparatively target-oriented evidence base. In gastric cancer, drug affinity responsive target stability (DARTS) and the cellular thermal shift assay (CETSA) supported EGFR engagement, while EGFR agonism partially reversed downstream STAT3 and ERK inhibition [52]. In NSCLC, p53 knockdown and miR-34a inhibition weakened acacetin-induced growth suppression and PD-L1 reduction, supporting a functional p53–miR-34a pathway [53]. Selective mitochondrial injury has also been reported in primary chronic lymphocytic leukemia cells relative to normal B lymphocytes [51]. In oral and head-and-neck squamous cell carcinoma, acacetin induced mitochondrial and caspase-dependent apoptosis, with functional evidence involving MAPK inhibition or muscarinic M3 receptor knockdown [54,55].

Linarin is an emerging constituent. In glioma, it reduced NF-κB/p65-associated signaling and increased p53-, p21-, Bax-, and caspase-related responses while suppressing xenograft growth [56]. More recent studies have linked linarin to HIF-1α/PD-L1 regulation and senescence-associated cell-cycle arrest [57,58]. In triple-negative breast cancer, linarin also reduced phosphorylated NF-κB p65 and MMP-9 while increasing Bax and caspase-3/7 activity, although relatively high concentrations were required, particularly in three-dimensional cultures [59]. Because these findings are recent and largely unreplicated, linarin should be regarded as promising rather than established.

Cirsimaritin has shown antiangiogenic, antiproliferative, and apoptosis-associated activity, but direct-target and in vivo evidence remain limited. The strongest plant-specific evidence derives from the *C. japonicum* var. *maackii* extract study involving VEGF, AKT, ERK, and endothelial tube formation [16]. Constituent-based studies have reported growth inhibition and ROS-associated apoptosis in lung squamous carcinoma and activity against hematologic malignancies [29,30]. Comparative flavonoid research has further suggested that methoxylation may influence tumor selectivity, although these structure–activity observations do not identify a direct molecular target for cirsimaritin [31].

### 2.5. Comparative Interpretation

The constituent landscape reveals a marked imbalance between chemical diversity and mechanistic depth. Pectolinarigenin and acacetin provide the strongest target-oriented evidence, whereas apigenin and luteolin provide the broadest hallmark coverage but the weakest plant specificity. Linarin and cirsimaritin remain emerging candidates, pectolinarin is less developed than its aglycone, and structurally distinctive phenylpropanoid glycosides remain promising but mechanistically unexplored lead compounds.

**Figure 1:**
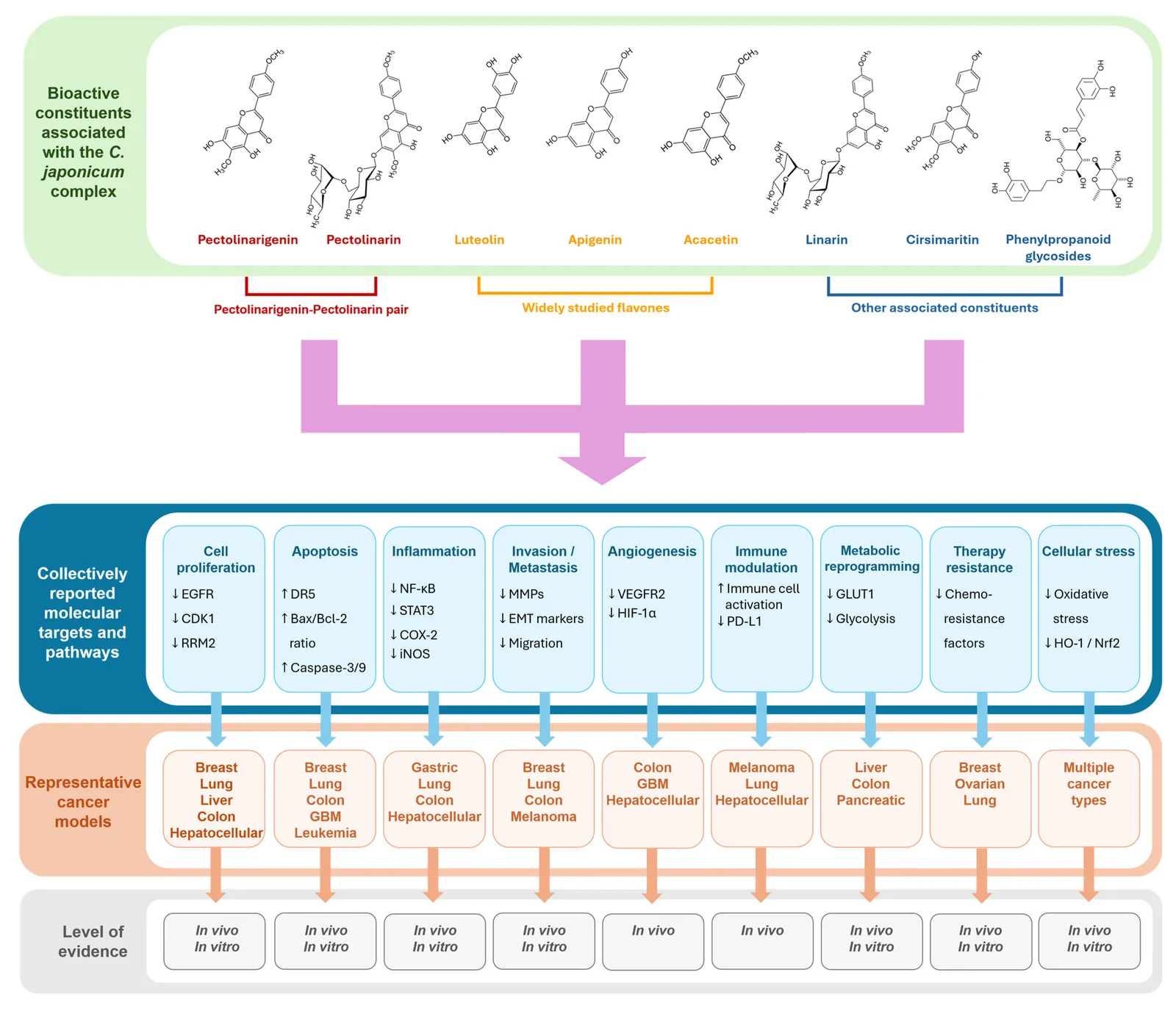
Bioactive constituents associated with *Cirsium japonicum* and collectively reported anticancer targets, pathways, cancer models, and levels of evidence. Chemical structures of representative bioactive constituents associated with *C. japonicum* and a collective summary of the molecular targets, biological pathways, cancer models, and evidence levels reported in the reviewed studies. The arrows indicate the organizational progression from the associated constituents to the collectively reported targets and pathways, representative cancer models, and corresponding levels of evidence. The molecular targets and pathways are presented collectively across the constituents and do not indicate that every constituent regulates every target shown. Compound-specific evidence and corresponding references are provided in Table 1 and Table 2 and discussed in the main text. GBM, glioblastoma; in vitro, cell-based evidence; in vivo, animal model evidence.

Representative preclinical studies are summarized in Table 1, whereas experimentally supported molecular targets and pathway dependencies are presented separately in Table 2. These comparisons indicate that the anticancer potential of *C. japonicum* cannot be inferred simply by aggregating all reported activities of its constituent flavonoids. Chemical abundance, glycoside–aglycone conversion, systemic and tumor exposure, direct target engagement, and reproducibility in clinically relevant models must be linked to biological efficacy before constituent-level findings can be attributed to standardized botanical preparations. Figure 1 summarizes the representative bioactive constituents of *C. japonicum*, their principal molecular targets and pathways, representative cancer models, and the relative strength of current experimental evidence.

## 3. Hallmark-Oriented Anticancer Mechanisms

### 3.1. Sustaining Proliferative Signaling and Evading Growth Suppressors

Several *C*. *japonicum*-associated constituents suppress cancer cell proliferation by interfering with receptor tyrosine kinase signaling, PI3K/AKT/mTOR and ERK pathways, cell-cycle regulators, and DNA replication machinery, all of which represent central drivers of uncontrolled tumor growth [6,7,65,66]. The strongest evidence is concentrated in pectolinarigenin and acacetin, whereas findings involving apigenin, luteolin, linarin, and cirsimaritin are generally more associative. The hallmark-oriented framework used throughout this review is summarized in Table 3.

Acacetin provides the clearest receptor-level evidence. EGFR is a central regulator of proliferative signaling in multiple human cancers and therefore represents an attractive therapeutic target [67]. In gastric cancer models, DARTS and CETSA supported acacetin–EGFR engagement, while EGFR agonism partially restored downstream STAT3 and ERK signaling. Acacetin also reduced colony formation and xenograft growth [52]. These findings support EGFR-dependent growth inhibition, although binding affinity, selectivity, and the precise interaction site remain undefined.

Pectolinarigenin acts at several levels of proliferative control. In glioblastoma, it increased autophagic flux and promoted RRM2 and CDK1 degradation, resulting in G2/M arrest. RRM2 overexpression partially reversed these effects, providing functional support for an RRM2–CDK1 dependency [20]. In bladder urothelial carcinoma, pectolinarigenin induced TOP2A-associated DNA damage, activated p53-related checkpoint signaling, and enhanced gemcitabine activity [21]. However, definitive classification as a TOP2A poison requires direct demonstration of cleavage complex stabilization.

Additional studies associate pectolinarigenin with PI3K/AKT/mTOR, ERK, PTEN, cyclins, and p21. These changes accompany reduced proliferation and G2/M arrest in hepatocellular carcinoma and suppression of malignant phenotypes in non-small-cell lung cancer [22,23]. Because most of these studies relied on phosphorylation and expression analyses, the pathways should be regarded as functionally associated rather than confirmed direct targets.

Growth suppressor reactivation has also been reported for acacetin and linarin. In NSCLC, p53 knockdown and miR-34a inhibition weakened acacetin-induced growth inhibition, supporting a functional p53–miR-34a axis [53]. Linarin reduced cyclin D1 and phosphorylated RB and increased p53 and p21 in glioma [56]. More recent evidence links linarin to cyclin A2-associated arrest and senescence, although these findings remain preliminary and lack in vivo or genetic rescue validation [58].

Apigenin and luteolin also inhibit proliferative signaling across several models, but their evidence is less specific to *C. japonicum*. Apigenin has been associated with PI3K/AKT/mTOR inhibition and G2/M arrest in hepatocellular and cervical cancer [32,38], while luteolin induces p53-associated growth suppression in colorectal cancer [47]. These studies broaden hallmark coverage but should remain classified as constituent-based evidence.

Overall, suppression of proliferative signaling is strongly supported for selected purified constituents but only weakly resolved for the plant itself. These observations are consistent with the established role of receptor tyrosine kinase and PI3K signaling in sustaining malignant proliferation [6]. The strongest mechanisms involve acacetin–EGFR engagement, pectolinarigenin-mediated RRM2–CDK1 degradation, TOP2A-associated DNA damage, and acacetin-mediated p53–miR-34a regulation. By contrast, recurrent changes in PI3K/AKT/mTOR, ERK, cyclins, and p21 are biologically consistent but often lack direct target or rescue validation.

### 3.2. Resisting Cell Death: Apoptosis, Autophagy, and Ferroptosis

Escape from regulated cell death is a defining characteristic of malignant progression and contributes directly to tumor persistence, metastatic dissemination, and therapeutic resistance [68]. In addition to suppressing apoptosis, cancer cells frequently exploit protective autophagy and antioxidant systems to survive metabolic stress and cytotoxic therapy. More recently, ferroptosis has emerged as a distinct form of regulated cell death characterized by iron-dependent lipid peroxidation and metabolic vulnerability [1,69,70].

Among the reported constituents, pectolinarigenin, luteolin, apigenin, acacetin, and linarin consistently induce apoptotic cell death across multiple tumor types, although the quality of mechanistic evidence differs considerably. Pectolinarigenin promotes mitochondrial apoptosis accompanied by Bax activation, Bcl-2 suppression, cytochrome *c* release, and caspase activation in nasopharyngeal carcinoma [18]. Similar mitochondrial responses have also been reported in hepatocellular carcinoma and glioblastoma [20,23]. While these studies consistently demonstrate apoptosis, mitochondrial dysfunction is generally interpreted as the initiating mechanism without identifying the primary molecular target responsible for triggering mitochondrial injury.

Luteolin provides the strongest evidence for receptor-mediated apoptosis. DR5 knockdown and DR5/Fc receptor blockade significantly reduced luteolin-induced caspase activation and apoptosis, demonstrating functional dependence on death receptor signaling [41]. Unlike many flavonoid studies that rely solely on altered Bax/Bcl-2 expression, this work incorporated genetic and pharmacological interventions, providing substantially stronger mechanistic support. Nevertheless, direct interaction between luteolin and DR5 has not been demonstrated, and whether DR5 activation results from direct receptor engagement or upstream cellular stress remains unresolved.

Acacetin also induces robust apoptosis but through a different mechanism. In chronic lymphocytic leukemia, acacetin selectively increased mitochondrial ROS, disrupted mitochondrial membrane potential, induced permeability transition, and activated caspase-dependent apoptosis in primary leukemia cells while producing relatively limited toxicity in normal B lymphocytes [51]. This comparison between malignant and normal primary cells provides one of the strongest demonstrations of tumor selectivity among the compounds reviewed. However, the molecular basis underlying this selectivity remains uncertain because the responsible mitochondrial target has not been identified.

Autophagy represents one of the most context-dependent mechanisms associated with *C. japonicum*-related compounds. Apigenin activates autophagy in hepatocellular carcinoma, but inhibition of ATG5 or pharmacological blockade of autophagy markedly enhances apoptosis and tumor suppression, indicating that autophagy functions primarily as a cytoprotective adaptation [32]. In contrast, pectolinarigenin promotes autophagic degradation of RRM2 and CDK1 in glioblastoma, resulting in irreversible cell-cycle arrest [20]. In bladder cancer, pectolinarigenin inhibits late autophagic flux while simultaneously enhancing gemcitabine-induced DNA damage [21]. These apparently divergent findings emphasize that autophagy cannot be uniformly classified as either tumor-promoting or tumor-suppressive. Instead, its biological significance depends on the stage of autophagic flux, cargo specificity, and cellular context, consistent with current concepts of autophagy biology [71,72].

Recent evidence further suggests that ferroptosis contributes to the anticancer activity of selected constituents. Luteolin increased intracellular Fe^2+^ accumulation, depleted glutathione, promoted lipid peroxidation, and induced HO-1-associated ferroptosis in clear-cell renal cell carcinoma. Ferrostatin-1 and iron-chelation experiments significantly attenuated cell death, providing functional evidence that ferroptosis contributes to the observed antitumor effect [43]. Similarly, *C. japonicum* var. *maackii*–mediated gold nanoparticles induced GPX4-associated antioxidant disruption and ferroptosis-like death in gastric cancer models [17]. However, because the latter study employed a nanoparticle formulation rather than a conventional plant extract, the observed effects cannot be attributed solely to phytochemicals derived from *C. japonicum*.

Collectively, the evidence indicates that *C. japonicum*-associated constituents regulate multiple forms of programmed cell death rather than apoptosis alone. Apoptotic signaling is consistently supported across numerous cancer models, whereas autophagy appears to function as either a survival mechanism or a mediator of tumor suppression depending on the biological context. Ferroptosis remains an emerging but particularly promising mechanism, especially for luteolin. Nevertheless, many studies continue to infer mechanisms from changes in apoptosis-related proteins or LC3-II expression without comprehensive flux analysis, rescue experiments, or direct target validation. Consequently, although regulation of cell death represents one of the strongest areas of anticancer evidence for *C. japonicum*-associated constituents, future investigations should incorporate comprehensive autophagic flux analyses, rescue experiments, and standardized approaches recommended by current autophagy guidelines [73].

### 3.3. Tumor-Promoting Inflammation

Chronic inflammation is widely recognized as a fundamental enabling characteristic of tumorigenesis because inflammatory mediators promote malignant transformation, tumor cell survival, angiogenesis, invasion, immune suppression, and therapeutic resistance. Persistent activation of NF-κB and STAT3 orchestrates cytokine production, epithelial–mesenchymal transition, recruitment of immunosuppressive cells, and establishment of a tumor-supportive microenvironment [1,74,75].

Several *C. japonicum*-associated constituents modulate inflammatory pathways, although the strength of evidence varies considerably. Among these, linarin, pectolinarigenin, and luteolin provide the most convincing mechanistic evidence, whereas most direct studies using *C. japonicum* extracts primarily demonstrate anti-inflammatory activity without establishing tumor-specific molecular mechanisms.

Linarin provides the most direct evidence linking inflammatory signaling to tumor suppression. In glioma models, linarin inhibited NF-κB activation by reducing nuclear p65 accumulation, accompanied by increased p53 expression, activation of caspase-dependent apoptosis, and suppression of xenograft growth [56]. Because NF-κB regulates numerous cytokines, survival genes, and anti-apoptotic proteins, these findings are biologically plausible. However, the study primarily relied on protein expression analyses and did not determine whether NF-κB inhibition represented the initiating molecular event or occurred secondary to cellular stress. Genetic rescue of NF-κB activity or pharmacological activation would strengthen the mechanistic conclusion.

Pectolinarigenin has been associated with inflammatory signaling primarily through STAT3 inhibition. In an immunocompetent 4T1 breast cancer metastasis model, pectolinarigenin reduced phosphorylation of STAT3 together with decreased expression of MMP-2 and MMP-9, increased TIMP2, and marked suppression of pulmonary metastasis [19]. Interestingly, increased infiltration of CD8^+^ T cells was also observed, suggesting that inhibition of STAT3 may influence both tumor cell behavior and the immune microenvironment. Nevertheless, constitutively active STAT3 rescue experiments were not performed, making it difficult to determine the relative contribution of STAT3 compared with other signaling pathways regulated by pectolinarigenin.

Luteolin extends these observations to the tumor microenvironment. In patient-derived glioblastoma stem cell models, luteolin suppressed IL-6/STAT3 signaling, reduced secretion of TGF-β1, inhibited polarization of macrophages toward an M2-like phenotype, and suppressed intracranial tumor growth [44]. Unlike conventional monoculture experiments, this study evaluated bidirectional communication between tumor cells and immune cells, providing a more physiologically relevant model of tumor-associated inflammation. However, macrophage polarization was characterized mainly using conventional M1/M2 markers, whereas recent studies indicate substantially greater myeloid heterogeneity within tumors. Single-cell transcriptomic analysis and macrophage depletion experiments would provide stronger evidence that remodeling of the immune microenvironment is required for the antitumor effects of luteolin.

Several additional constituents influence inflammatory mediators, including PI3K/AKT-, MAPK-, and cytokine-associated pathways. However, in many studies inflammatory proteins were measured alongside apoptosis or oxidative stress without demonstrating that inflammation itself was required for tumor suppression. Consequently, reductions in IL-6, TNF-α, COX-2, iNOS, or NF-κB activity should generally be interpreted as associated biological responses rather than validated therapeutic targets unless supported by functional rescue experiments.

An important distinction should also be made between anti-inflammatory pharmacology and anticancer immunomodulation. Suppression of inflammatory cytokines may inhibit tumor-promoting inflammation, but excessive suppression could also impair antigen presentation or cytotoxic immune responses. Therefore, anti-inflammatory activity alone cannot be assumed to improve antitumor immunity. This distinction becomes particularly important when interpreting studies performed in inflammatory disease models, which may not accurately predict responses within the tumor microenvironment.

Overall, current evidence supports inflammation as one of the major biological processes regulated by *C. japonicum*-associated constituents, particularly through NF-κB-, STAT3-, and IL-6-associated pathways. However, mechanistic depth remains heterogeneous. Linarin provides the strongest evidence for NF-κB-associated regulation, pectolinarigenin links STAT3 inhibition with reduced metastatic potential, and luteolin extends inflammatory regulation to macrophage remodeling within the tumor microenvironment. Despite these advances, direct target validation, immune cell dependency studies, and immunocompetent tumor models remain relatively uncommon. Consequently, inflammatory signaling should currently be regarded as moderately supported at the constituent level but only weakly established for standardized *C. japonicum* preparations.

### 3.4. Activating Invasion and Metastasis

Metastasis remains the principal cause of cancer-related mortality and involves epithelial–mesenchymal transition (EMT), extracellular matrix degradation, tumor cell migration, intravasation, survival in the circulation, extravasation, and colonization of distant organs [76,77]. These processes are regulated by multiple signaling pathways including PI3K/AKT, STAT3, TGF-β/SMAD, HIF-1α, CXCR4, integrins, and matrix metalloproteinases.

Among *C. japonicum*-associated constituents, pectolinarigenin, luteolin, apigenin, and linarin demonstrate the most consistent antimetastatic activity.

Pectolinarigenin currently provides the strongest evidence for suppression of metastatic progression. In an immunocompetent 4T1 breast cancer model, pectolinarigenin significantly reduced pulmonary metastatic burden while decreasing STAT3 phosphorylation, MMP-2, and MMP-9 expression and increasing TIMP2 [19]. The use of a spontaneous lung metastasis model represents a notable strength because it evaluates metastatic dissemination rather than migration in cultured cells. However, constitutively active STAT3 rescue experiments were not performed, and therefore STAT3 should be regarded as a major associated pathway rather than a definitively validated primary target.

Additional studies indicate that pectolinarigenin suppresses EMT-associated phenotypes through PTEN-dependent inhibition of PI3K/AKT signaling. Reduced expression of N-cadherin, vimentin, and Snail together with restoration of E-cadherin has been observed in non-small-cell lung cancer, accompanied by decreased migration and invasion [22]. Similar effects have been reported in hepatocellular carcinoma and cervical cancer [23,78]. Although these findings consistently support inhibition of EMT, most studies rely on wound-healing or Transwell assays and therefore evaluate migratory potential rather than complete metastatic competence.

Luteolin has been linked to metastasis through several complementary mechanisms. In colorectal cancer, luteolin inhibited AhR-dependent CXCR4 expression, reduced MMP-2 and MMP-9, and suppressed experimental lung metastasis [42]. In addition, luteolin reduced integrin β1-associated signaling and VEGFA expression, thereby impairing both invasive behavior and angiogenic support [48]. Together, these findings suggest that luteolin may interfere with both tumor cell migration and formation of a permissive metastatic microenvironment. Nevertheless, direct biochemical interaction with AhR or integrin β1 has not been demonstrated, and additional target engagement studies are required.

Apigenin also inhibits metastatic phenotypes in several cancer models, largely through suppression of PI3K/AKT signaling, EMT-associated transcription factors, and MMP expression [38]. However, most evidence derives from in vitro migration assays, with relatively limited validation in spontaneous or orthotopic metastasis models. Consequently, apigenin currently provides broader mechanistic support than direct evidence for inhibition of metastatic dissemination.

Recent studies further suggest that linarin may suppress metastatic adaptation through regulation of the HIF-1α/PD-L1 axis. In colorectal cancer, linarin reduced migration and invasion while decreasing both HIF-1α and PD-L1 expression, and overexpression of either molecule partially reversed these effects [57]. Because hypoxia contributes to EMT, immune evasion, and metastatic colonization, these observations may extend beyond checkpoint regulation alone. Nevertheless, the experiments were performed primarily in xenograft models lacking intact adaptive immunity, limiting interpretation of PD-L1-mediated immune escape.

Despite encouraging preclinical findings, several methodological limitations remain. Many studies evaluate EMT using changes in E-cadherin, N-cadherin, vimentin, or Snail expression together with wound-healing assays. While these measurements are widely accepted as indicators of migratory potential, they cannot fully reproduce the complexity of metastatic progression, which requires intravasation, survival in circulation, immune evasion, colonization of distant organs, and interactions with stromal and immune cells. Similarly, suppression of MMP expression does not necessarily establish reduced metastatic competence without appropriate in vivo validation.

Overall, inhibition of invasion and metastasis represents one of the more consistently supported biological activities of *C. japonicum*-associated constituents. Pectolinarigenin provides the strongest evidence through spontaneous metastasis models and STAT3-associated regulation, whereas luteolin broadens mechanistic coverage by targeting AhR–CXCR4, integrin β1, and angiogenesis-associated pathways. Apigenin and linarin provide additional support but rely predominantly on pathway association and in vitro migration assays. Accordingly, the current evidence supports moderate-to-strong constituent-level antimetastatic activity, whereas direct evidence for standardized *C. japonicum* preparations remains limited. Future studies should prioritize orthotopic and spontaneous metastasis models, lineage tracing approaches, and direct target validation experiments to establish causal relationships between molecular signaling and metastatic suppression.

### 3.5. Suppressing Angiogenesis and Hypoxic Adaptation

Angiogenesis is essential for sustained tumor growth and metastatic dissemination because expanding tumors require a continuous supply of oxygen and nutrients. Tumor hypoxia stabilizes HIF-1α, thereby inducing VEGF expression, metabolic adaptation, epithelial–mesenchymal transition, and therapeutic resistance [79,80,81].

Several *C. japonicum*-associated constituents exhibit antiangiogenic activity, although the supporting evidence varies substantially in mechanistic depth. Luteolin, apigenin, cirsimaritin, and linarin provide the strongest evidence, whereas direct studies using *C. japonicum* extracts remain relatively limited.

Direct plant-specific evidence is available from *C. japonicum* var. *maackii*. An ethanol extract and its major constituent, cirsimaritin, inhibited endothelial cell proliferation and tube formation while reducing VEGF-, AKT-, and ERK-associated signaling [16]. Because endothelial cells rather than tumor cells were primarily investigated, the study demonstrates antiangiogenic potential rather than direct inhibition of tumor progression. Nevertheless, it provides one of the few mechanistic studies performed using a genuine *C. japonicum* preparation instead of commercially available flavonoids.

Among purified constituents, luteolin has the most comprehensive evidence. Luteolin suppresses VEGF-associated angiogenic signaling through multiple mechanisms, including inhibition of VEGFR2 activation, integrin β1-associated signaling, and downstream AKT and ERK pathways, consistent with current concepts of vascular normalization and angiogenic regulation [48,49]. Functional inhibition of integrin β1 significantly attenuated vascular signaling and enhanced radiosensitivity, suggesting that luteolin interferes with both angiogenesis and tumor adaptation to therapeutic stress [48]. However, despite convincing functional evidence, direct biochemical interaction between luteolin and integrin β1 or VEGFR2 has not been established.

Apigenin has likewise demonstrated antiangiogenic activity in several experimental systems. Reduced VEGF expression and suppression of angiogenesis-associated signaling have been reported in cervical and lung cancer models [38,82]. Most studies, however, rely on measurements of VEGF expression or endothelial cell behavior without directly evaluating vascular perfusion, vessel maturation, or functional blood flow within tumors. Consequently, the evidence is best interpreted as inhibition of angiogenic signaling rather than definitive vascular normalization.

Hypoxic adaptation has received increasing attention in recent years. Linarin represents the strongest example within the current literature. In colorectal cancer, linarin promoted degradation of HIF-1α, reduced PD-L1 expression, inhibited proliferation and invasion, and these effects were partially reversed by HIF-1α or PD-L1 overexpression [57]. These findings suggest that linarin influences both hypoxia-associated tumor adaptation and immune checkpoint regulation. Nevertheless, the experiments were performed primarily in immunodeficient xenograft models, preventing evaluation of adaptive immune responses and limiting conclusions regarding immune checkpoint modulation.

Several additional constituents indirectly influence hypoxia-related pathways through suppression of PI3K/AKT, STAT3, or inflammatory signaling. However, changes in HIF-1α expression frequently occur secondary to reduced oxidative stress or inhibition of upstream oncogenic signaling. Therefore, decreased HIF-1α should not automatically be interpreted as evidence of direct hypoxia targeting unless supported by mechanistic rescue or target engagement experiments.

Another important limitation is that many antiangiogenic studies continue to rely on endothelial tube-formation assays. Although these experiments are valuable screening tools, they cannot reproduce the structural and functional complexity of tumor vasculature. Mature tumor vessels interact extensively with pericytes, extracellular matrix, inflammatory cells, and hypoxic gradients, features that are largely absent from conventional in vitro assays. Future studies should therefore incorporate orthotopic tumor models, vascular imaging, microvessel density quantification, perfusion analyses, and spatial characterization of hypoxic regions.

Overall, inhibition of angiogenesis and hypoxic adaptation is moderately to strongly supported at the constituent level, with luteolin providing the broadest mechanistic evidence and linarin offering particularly interesting links between HIF-1α, PD-L1, and tumor adaptation. Direct plant-specific evidence remains comparatively limited but supports the biological plausibility of antiangiogenic activity in *C. japonicum*. Current evidence also indicates that angiogenesis, hypoxia, inflammation, and immune regulation are highly interconnected rather than independent biological processes. Accordingly, future investigations should evaluate these pathways within integrated tumor microenvironment models rather than as isolated signaling cascades.

### 3.6. Avoiding Immune Destruction and Remodeling the Tumor Microenvironment

Cancer immunosurveillance constitutes a critical barrier against tumor progression, whereas successful tumors develop multiple mechanisms to evade immune destruction and establish an immunosuppressive tumor microenvironment [83,84,85,86]. Tumors evade immune elimination by reducing antigen presentation, increasing immune checkpoint signaling, excluding cytotoxic lymphocytes, and recruiting suppressive myeloid populations. Hypoxia, STAT3 activation, TGF-β signaling, and inflammatory cytokines further reinforce this immunosuppressive microenvironment [1,85,86]. The clinical success of cancer immunotherapy further highlights immune evasion as a central hallmark of cancer [85].

Pectolinarigenin provides the most relevant evidence from an immunocompetent model. In 4T1 breast cancer, it reduced pulmonary metastasis and increased CD8^+^ T-cell abundance in blood and lung tissue, together with suppression of STAT3 phosphorylation and MMP-2/9 expression [19]. The intact immune system and metastatic model strengthen the biological relevance of these observations. However, increased CD8^+^ T-cell numbers do not prove tumor-specific cytotoxic activity, and the study did not include CD8 depletion, cytokine production, granzyme B analysis, or T-cell exhaustion markers. Pectolinarigenin should therefore be described as promoting CD8^+^ T-cell recruitment associated with metastatic suppression, rather than as definitively restoring adaptive antitumor immunity.

Linarin has been linked to immune-checkpoint-associated regulation through the HIF-1α/PD-L1 axis. In colorectal cancer, linarin reduced HIF-1α stability and PD-L1 expression, and overexpression of either protein partially reversed its effects on proliferation, apoptosis, migration, and invasion [57]. These rescue experiments provide relatively strong evidence for tumor-cell-intrinsic pathway dependency. Nevertheless, the xenograft model did not permit full evaluation of PD-1/PD-L1-mediated T-cell suppression. Thus, this study supports regulation of an immune-evasion-associated signaling axis, but not yet functional checkpoint inhibition.

Luteolin extends the evidence to tumor–myeloid interactions. In glioblastoma models, luteolin reduced IL-6/STAT3 and TGF-β1/SMAD3 signaling, inhibited glioma stem cell growth and invasion, and suppressed macrophage polarization toward an M2-like phenotype [44]. This study is important because it evaluated communication between malignant and immune cells rather than tumor-cell-autonomous signaling alone. However, conventional M1/M2 classification incompletely represents the diversity of tumor-associated macrophages, and macrophage depletion or lineage-specific rescue was not performed.

Early direct studies of *C. japonicum* flavonoid fractions also reported increased cellular and humoral immune indices in tumor-bearing mice [14]. Although these findings suggest host-mediated activity, the measured endpoints do not identify the immune populations responsible for tumor control and predate current concepts of checkpoint regulation, T-cell exhaustion, and myeloid heterogeneity. They therefore provide preliminary plant-specific evidence rather than mechanistically resolved immunotherapy data.

Overall, immune modulation is less firmly established than effects on proliferation, apoptosis, or metastasis. Pectolinarigenin provides the strongest evidence for immune cell recruitment in an immunocompetent model, linarin regulates a hypoxia-associated PD-L1 axis, and luteolin alters glioma–macrophage communication. However, none has yet demonstrated durable antigen-specific immune memory or consistent enhancement of immune checkpoint blockade. Future studies should employ syngeneic or humanized models, immune cell depletion, spatial immune profiling, and combinations with PD-1/PD-L1 inhibitors. Current evidence is therefore best classified as emerging to moderately supported at the constituent level and limited for standardized *C. japonicum* preparations.

### 3.7. Deregulating Cellular Energetics and Redox Homeostasis

Metabolic reprogramming enables cancer cells to maintain ATP production, biosynthesis, and redox homeostasis during rapid proliferation and therapeutic stress. In addition to enhanced glycolysis, malignant cells dynamically reprogram mitochondrial metabolism, glutamine utilization, lipid metabolism, and antioxidant defense [8,87].

Among the reported constituents, apigenin, luteolin, and acacetin provide the strongest evidence for targeting metabolic adaptation. Apigenin suppresses GLUT1-associated glucose utilization and PI3K/AKT signaling, thereby enhancing radiosensitivity in laryngeal carcinoma [40]. In hepatocellular carcinoma, apigenin also attenuated Nrf2-dependent antioxidant defense and ATG7-mediated protective autophagy, restoring sensitivity to doxorubicin [33,35]. These studies indicate that apigenin functions primarily as a metabolic and redox sensitizer rather than a direct cytotoxic agent.

Luteolin has generated the strongest evidence for ferroptosis-associated metabolic vulnerability. In clear-cell renal cell carcinoma, luteolin increased intracellular Fe^2+^ accumulation, depleted glutathione, promoted lipid peroxidation, and induced HO-1-associated ferroptosis, effects that were significantly attenuated by ferrostatin-1 and iron chelation [43]. Functional rescue experiments provide considerably stronger support than studies relying solely on ROS measurements. Nevertheless, direct interaction between luteolin and HO-1 has not been established, and the observed effects should be interpreted as pathway-associated rather than target-specific.

Acacetin preferentially disrupted mitochondrial function in primary chronic lymphocytic leukemia cells while producing substantially less injury in normal B lymphocytes [51]. Increased mitochondrial ROS, collapse of membrane potential, permeability transition, and cytochrome *c* release suggest that malignant mitochondria may represent a selective metabolic vulnerability. However, the molecular determinants responsible for this apparent selectivity remain unknown.

Direct plant-specific evidence for metabolic regulation remains limited. *C. japonicum var. maackii*-mediated gold nanoparticles promoted ferroptosis-like death through ROS generation, iron accumulation, and GPX4-associated antioxidant disruption [17]. Because nanoparticle formulation itself substantially influences intracellular uptake and oxidative stress, these findings cannot be directly extrapolated to conventional *C. japonicum* extracts.

Despite encouraging results, current evidence has important limitations. Most studies evaluate ROS production, mitochondrial membrane potential, GLUT1 expression, or antioxidant proteins without directly measuring metabolic flux. Stable-isotope tracing, Seahorse extracellular flux analysis, metabolomics, lipidomics, and quantitative assessment of ATP turnover remain largely absent. Consequently, many reports describe metabolic-associated signaling rather than comprehensive metabolic reprogramming.

Overall, modulation of cellular energetics and redox homeostasis is moderately supported at the constituent level, with particularly strong evidence for luteolin-induced ferroptosis and apigenin-mediated reversal of metabolic therapy resistance. In contrast, direct evidence for standardized *C. japonicum* preparations remains limited, and whether sufficient concentrations of active constituents reach tumor tissue to reproduce these metabolic effects has not been established. Future studies integrating pharmacokinetics, metabolomics, isotope tracing, and target engagement analyses will be essential to define the true translational significance of these findings.

### 3.8. Therapy Resistance, Senescence, and Phenotypic Plasticity

Therapeutic resistance remains one of the greatest challenges in oncology and frequently develops through non-mutational adaptive mechanisms rather than acquisition of new genetic alterations. Cancer cells exposed to chemotherapy, radiotherapy, or targeted therapy activate protective autophagy, antioxidant defenses, hypoxia-associated signaling, metabolic reprogramming, phenotypic plasticity, and senescence-like states that promote survival under therapeutic stress [1,8].

Among *C. japonicum*-associated constituents, apigenin currently provides the strongest evidence for overcoming acquired drug resistance. In doxorubicin-resistant hepatocellular carcinoma, apigenin inhibited PI3K/AKT/Nrf2 signaling, reduced ATG7-dependent protective autophagy, increased intracellular doxorubicin accumulation, and restored chemosensitivity [33,35]. Functional modulation of miR-101 and miR-520b further supported the involvement of antioxidant and autophagic pathways. Although these studies provide convincing mechanistic evidence, most originate from a closely related resistant HCC model, and broader validation across independent tumor systems remains necessary.

Pectolinarigenin also demonstrates considerable potential as a chemosensitizing agent. In bladder urothelial carcinoma, pectolinarigenin induced DNA damage, impaired late autophagic flux, and significantly enhanced the antitumor activity of gemcitabine [21]. Rather than acting solely as a cytotoxic compound, pectolinarigenin appears to disrupt adaptive survival mechanisms that normally limit chemotherapy-induced cell death. However, formal synergy analysis, pharmacokinetic compatibility, and normal-tissue toxicity have not yet been comprehensively evaluated.

Several constituents have likewise shown radiosensitizing activity. Apigenin increased radiosensitivity through suppression of GLUT1-associated metabolic adaptation [40], whereas luteolin enhanced radiation-induced apoptosis through ROS-dependent activation of the p38/caspase pathway and inhibition of integrin β1-associated signaling [46,48]. Collectively, these findings suggest that *C. japonicum*-associated flavonoids may be more valuable as adjuvant agents than as stand-alone cytotoxic drugs.

Evidence for phenotypic plasticity and senescence is comparatively recent. Linarin induced G0/G1 arrest, increased senescence-associated β-galactosidase activity, and reduced cyclin A2, cyclin B1, and CHEK1 expression in non-small-cell lung cancer cells [58]. While these observations suggest activation of a senescence-like program, it remains unclear whether the resulting growth arrest is irreversible or whether senescent cells retain the capacity to promote tumor recurrence through the senescence-associated secretory phenotype (SASP). Long-term clonogenic recovery, SASP characterization, and immune-mediated clearance have not yet been investigated.

Tumor cell plasticity has also been implicated in studies of luteolin. Suppression of IL-6/STAT3 signaling and disruption of communication between glioma stem cells and tumor-associated macrophages suggest that luteolin may interfere with adaptive stem-like phenotypes as well as the supportive tumor microenvironment [44]. However, direct evidence demonstrating reduced cancer stem cell frequency or long-term prevention of tumor recurrence remains limited.

Overall, current evidence indicates that *C. japonicum*-associated constituents are particularly effective at disrupting adaptive survival mechanisms rather than simply inducing acute cytotoxicity. Apigenin provides the strongest support for reversal of chemotherapy resistance, pectolinarigenin enhances the activity of DNA-damaging agents through modulation of autophagy, luteolin functions as a promising radiosensitizer, and linarin introduces the emerging concept of senescence-associated tumor suppression. Nevertheless, most studies remain confined to preclinical models, and relatively few evaluate long-term recurrence, treatment durability, or clinically relevant combination strategies. Consequently, therapy sensitization is currently one of the most translationally promising but still incompletely validated applications of *C. japonicum*-associated constituents. Future investigations should prioritize standardized combination protocols, biomarker-guided patient selection, pharmacokinetic optimization, and long-term assessment of relapse following treatment withdrawal. Collectively, these findings indicate that evidence strength varies substantially among individual hallmarks and compounds, as summarized in Table 4.

## 4. Critical Challenges and Future Perspectives

**Figure 2:**
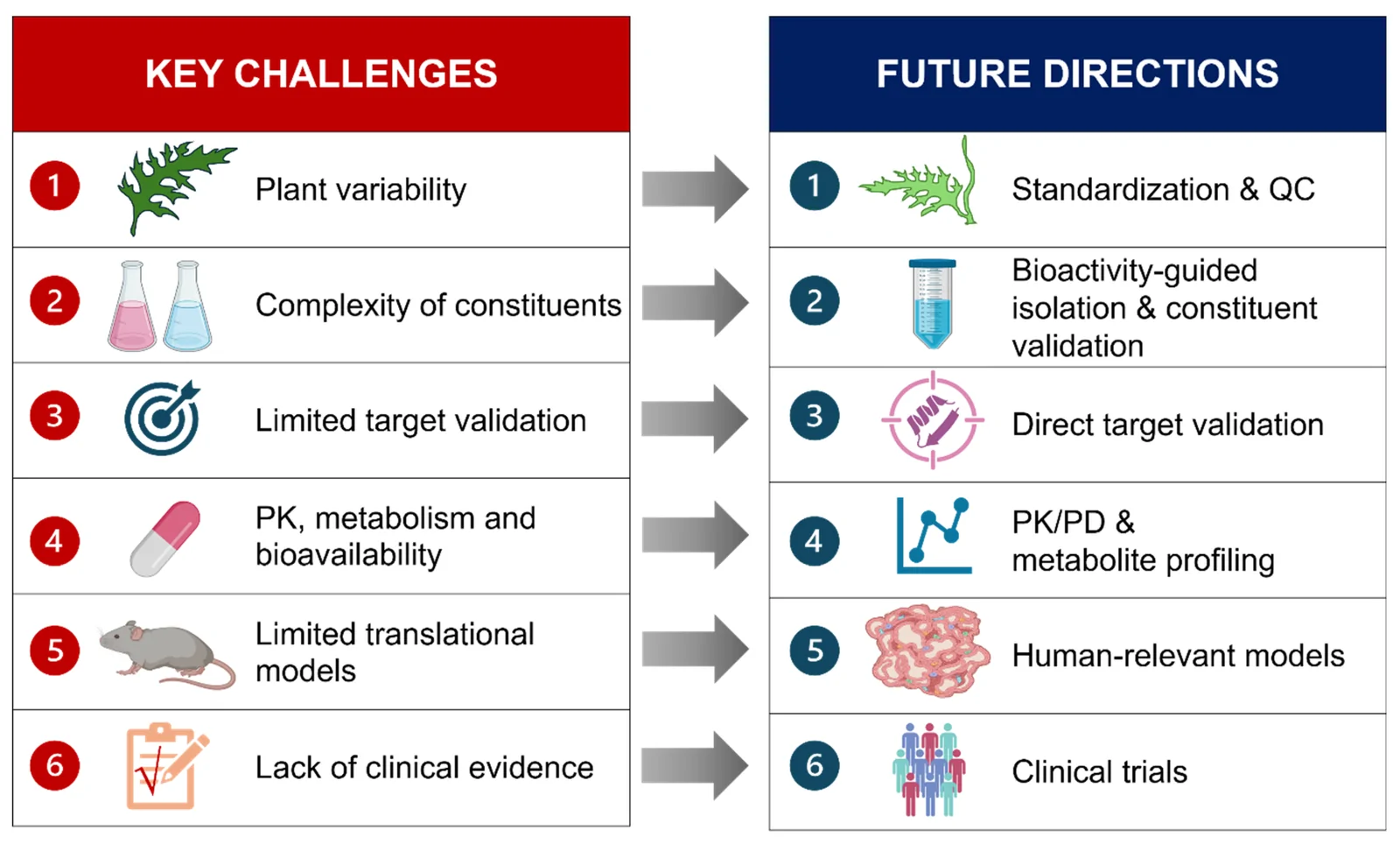
Key challenges and future research priorities for the translation of *C. japonicum* and its associated constituents into cancer therapeutics. Created in BioRender. Kim, K. (2026) https://BioRender.com/lfy1s7n.

Although accumulating evidence supports the anticancer potential of *C*. *japonicum* and its associated phytochemicals, significant challenges remain before these findings can be translated into clinically meaningful applications. Throughout this review, we identified substantial differences in the strength of mechanistic evidence, experimental design, pharmacological validation, and translational relevance among published studies. Rather than reflecting weaknesses of the field, these limitations define the priorities for future investigation. Similar translational challenges have been recognized broadly in natural-product pharmacology and cancer drug development, where mechanistic validation, pharmacokinetics, and clinically relevant disease models remain major obstacles to successful translation [1,8]. The principal methodological and translational challenges identified throughout this review, together with the corresponding future research priorities, are summarized in Figure 2.

### 4.1. Distinguishing Plant Pharmacology from Constituent Pharmacology

One of the most important observations emerging from this review is that the anticancer evidence for *C. japonicum* is considerably weaker than that for its individual phytochemicals. Most mechanistic studies have been performed using commercially available compounds or constituents isolated from botanical species other than *C. japonicum*, whereas relatively few investigations have evaluated standardized *C. japonicum* preparations. Consequently, molecular mechanisms identified for apigenin, luteolin, acacetin, or pectolinarigenin cannot automatically be attributed to the medicinal plant itself.

This distinction is particularly important because phytochemical composition varies substantially according to plant origin, extraction method, harvest season, processing conditions, and post-harvest storage. Moreover, glycosides and aglycones may exhibit distinct pharmacological properties and should not automatically be considered pharmacologically equivalent. Future studies should therefore directly compare standardized extracts, purified compounds, constituent-depleted fractions, and chemically reconstituted mixtures. Such comparative approaches would help distinguish pharmacological effects attributable to individual compounds from those resulting from additive or synergistic interactions within the botanical extract. The principal methodological limitations of the current literature and the corresponding experimental strategies required to address them are summarized in Table 5.

### 4.2. From Correlative Signaling to Target-Based Pharmacology

Another major limitation of the current literature is the predominance of pathway-association studies. In many reports, alterations in PI3K/AKT, STAT3, NF-κB, MAPK, VEGF, or HIF-1α signaling are interpreted as molecular mechanisms solely because changes in phosphorylation or protein expression are observed after treatment. However, these alterations frequently represent downstream adaptive responses rather than primary pharmacological targets.

Encouragingly, several recent studies have begun to address this limitation. DARTS and CETSA supported EGFR engagement by acacetin, RRM2 overexpression demonstrated functional dependency during pectolinarigenin treatment, DR5 silencing established receptor-mediated apoptosis induced by luteolin, and HIF-1α/PD-L1 overexpression confirmed pathway involvement during linarin treatment. These studies represent an important transition from descriptive signaling analysis toward mechanism-based pharmacology.

Nevertheless, such examples remain the exception rather than the rule. Future research should increasingly incorporate modern target identification technologies, including thermal proteome profiling, affinity-based chemoproteomics, CRISPR screening, spatial proteomics, and quantitative target engagement assays. These approaches will facilitate discrimination between direct target engagement and secondary signaling responses while providing a stronger mechanistic foundation for rational drug development and biomarker-guided precision oncology.

### 4.3. Translational Barriers Beyond Mechanism

Strong mechanistic evidence alone does not ensure clinical applicability. Most studies reviewed here were performed in conventional two-dimensional cancer cell lines or immunodeficient xenograft models and therefore incompletely represent tumor heterogeneity, stromal interactions, immune surveillance, and metabolic adaptation. Likewise, pharmacokinetic characterization, tissue distribution, bioavailability, and clinically achievable exposure remain largely unexplored for most constituents. Similar translational limitations have been widely recognized across flavonoid pharmacology, where poor oral bioavailability, rapid metabolism, and limited tumor exposure frequently reduce clinical applicability.

These issues are particularly relevant for flavonoids, many of which undergo extensive intestinal metabolism, glucuronidation, sulfation, and rapid systemic clearance. Similarly, glycosides such as pectolinarin may exhibit pharmacological behavior that differs substantially from that of their aglycones. Consequently, pharmacologically active circulating metabolites rather than parent compounds may contribute substantially to the biological effects observed in vivo.

Future investigations should integrate quantitative pharmacokinetics with metabolomics, tissue distribution analysis, target engagement, and pharmacodynamic biomarkers. In parallel, advanced experimental models—including patient-derived organoids, orthotopic tumors, syngeneic and humanized mouse models, spatial transcriptomics, and single-cell multi-omics—will provide a more clinically relevant framework for evaluating therapeutic efficacy. The translational positioning, principal unresolved limitations, and priority development needs of the major *C. japonicum*-associated materials are summarized in Table 6. Such models are increasingly recognized as essential tools for improving translational predictability in oncology [4,5].

### 4.4. Future Perspectives

Collectively, the available evidence suggests that *C. japonicum* should not be viewed simply as a source of broadly cytotoxic flavonoids. Instead, it represents a pharmacologically diverse platform containing multiple bioactive constituents that modulate interconnected hallmarks of cancer through distinct but partially overlapping mechanisms. Importantly, different constituents appear to possess complementary pharmacological profiles: pectolinarigenin exhibits the strongest evidence for cell-cycle regulation and chemosensitization, acacetin demonstrates comparatively advanced target validation, luteolin displays the broadest hallmark coverage, apigenin is particularly effective at overcoming therapy resistance, whereas linarin represents an emerging regulator of hypoxia-associated adaptation and immune signaling. Rather than functioning as pharmacologically redundant flavonoids, these constituents appear to occupy complementary mechanistic niches across different hallmarks of cancer.

Future research should therefore shift from simply identifying additional anticancer activities toward defining which constituent, at what exposure, in which molecular subtype, and under which therapeutic context is most likely to provide clinical benefit. Biomarker-guided strategies incorporating molecular subtype, metabolic phenotype, immune status, and treatment history may ultimately allow *C. japonicum*-derived compounds to be positioned within precision oncology rather than as generalized botanical therapeutics. Integrating standardized phytochemistry, quantitative pharmacokinetics, target-based pharmacology, systems biology, and clinically relevant disease models will be essential for translating these promising preclinical observations into evidence-based precision oncology. Ultimately, future progress will depend not only on discovering additional biological activities but also on establishing reproducible exposure–target–response relationships that support clinical development. An integrated overview of the compound–hallmark relationships and future translational priorities is presented in Table 7.

## 5. Conclusions

The Hallmarks of Cancer framework indicates that constituents associated with the *C. japonicum* complex influence multiple processes relevant to tumor progression, but the strength and specificity of the supporting evidence vary substantially. Direct plant-specific studies remain limited, whereas most mechanistic evidence has been obtained using purified compounds that were commercially sourced or isolated from other botanical species. Constituent-level activity therefore cannot be assumed to represent the pharmacology of standardized *C. japonicum* preparations.

Overall, the *C. japonicum* complex should be regarded as a source of potentially useful anticancer lead compounds rather than an established botanical therapy. Its principal research value lies in linking clearly defined botanical materials and constituents to experimentally validated targets, achievable exposure, and reproducible antitumor effects. Until these relationships are established, therapeutic interpretations should remain preclinical and should clearly distinguish botanical occurrence, constituent-based activity, and direct plant-specific evidence.

## Figures and Tables

**Table 1:** Preclinical evidence for *C. japonicum* and its associated constituents.

Compound/Material	Cancer Model and Study System	Principal Findings	Mechanistic Validation	In Vivo Evidence	Evidence Category	Key Reference
Total flavone preparation from *C. japonicum* (FLCJ), pectolinarin, and 5,7-dihydroxy-6,4′-dimethoxyflavone	S180- and H22-tumor-bearing mice	Inhibition of implanted tumor growth and enhancement of cellular and humoral immune responses; FLCJ showed greater activity than either isolated flavone	Immune response assessment; direct molecular targets not examined	Tumor-growth inhibition	Direct plant-specific	[14]
Pectolinarin and 5,7-dihydroxy-6,4′-dimethoxyflavone isolated from *C. japonicum*	Extract analysis; S180- and H22-tumor-bearing mice	Isolation and RP-HPLC quantification of the two flavones in methanol, ethanol, and aqueous extracts; inhibition of S180 tumor growth and prolongation of survival in H22-bearing mice	Chemical isolation and quantitative RP-HPLC analysis; direct molecular targets not examined	Tumor-growth inhibition and prolonged survival	Direct plant-specific	[15]
Phenylpropanoid glycosides isolated from *C. japonicum*	MCF-7, U87, HCT116, and A549 cells	Low-micromolar cytotoxicity for selected compounds	Direct targets and cell-death mechanisms not examined	Not tested	Direct plant-specific	[13]
*C. japonicum* var. *maackii* extract and cirsimaritin	MDA-MB-231 cells and HUVECs	Reduced endothelial cell viability, VEGF production, AKT/ERK phosphorylation, and tube formation	Pathway-associated and endothelial functional assays	Not tested	Direct plant-specific	[16]
CJ-AuNPs	AGS gastric cancer cells and xenograft model	Increased ROS, Fe^2+^ accumulation, and lipid peroxidation; impaired GPX4-associated antioxidant defense	Ferroptosis-associated assays; botanical and gold-core effects not separated	Reduced xenograft growth	Plant-derived nanomaterial	[17]
Pectolinarigenin	C666-1 nasopharyngeal carcinoma cells and xenograft model	ROS-associated mitochondrial apoptosis and reduced tumor growth	ROS and apoptosis-related interventions; direct target not established	Reduced xenograft growth	Constituent-based	[18]
	4T1, MDA-MB-231, and MCF-7 cells; pulmonary metastasis model	Reduced STAT3 and MMP-2/9 signaling and pulmonary metastasis; increased CD8^+^ T-cell recruitment	Pathway-associated analyses	Reduced pulmonary metastasis	Constituent-based	[19]
	Glioblastoma cells; subcutaneous and orthotopic models	Autophagic degradation of RRM2 and CDK1 and induction of G2/M arrest	RRM2 overexpression rescue and autophagic flux analysis	Reduced tumor growth	Constituent-based	[20]
	Bladder urothelial carcinoma cells and xenograft model	DNA damage, altered autophagic flux, and enhanced gemcitabine activity	Target/pathway and combination analyses; direct TOP2A poisoning not established	Positive single-agent and combination effects	Constituent-based	[21]
	A549 and Calu-3 NSCLC cells	Increased PTEN expression, reduced PI3K/AKT signaling, and reversal of EMT-associated phenotypes	PTEN inhibitor and AKT activator interventions	Not tested	Constituent-based	[22]
	HCC cells and xenograft model	Reduced proliferation and motility; induced apoptosis and G2/M arrest through PI3K/AKT/mTOR/ERK-associated signaling	Primarily pathway-associated analyses; direct target not established	Reduced xenograft growth	Constituent-based	[23]
	Pectolinarigenin isolated from *Tiliacora triandra*; triple-negative breast cancer cells	G0/G1 arrest, apoptosis, and MAPK signaling inhibition	Cell-cycle, apoptosis, and pathway analyses; direct target not established	Not tested	Constituent-based	[24]
	AGS gastric cancer xenograft model	Reduced tumor growth and altered tumor-associated protein expression	Tumor-protein expression profiling; primary target not established	Reduced xenograft growth	Constituent-based	[25]
Pectolinarin	Lung, colorectal, and melanoma cell lines	Cytotoxicity with structure-dependent differences	Direct targets and cell-death mechanisms not established	Not tested	Constituent-based	[26]
	PC12 cells exposed to cellular stress	ER-stress-associated responses involving ATF6, PERK–eIF2α, and IRE1	Pathway-associated analyses	Not tested	Contextual mechanistic evidence	[27]
	Rheumatoid arthritis fibroblast-like synoviocytes	Reduced proliferation and inflammation and induced apoptosis through PI3K/AKT-associated signaling	Pathway-associated analyses	Not tested	Contextual mechanistic evidence	[28]
Cirsimaritin	Lung squamous carcinoma cells	Growth inhibition and ROS-associated apoptosis	ROS and apoptosis-related analyses; direct target not established	Not tested	Constituent-based	[29]
	Hematologic malignancy models	Antiproliferative and apoptosis-associated activity	Primarily expression- and viability-based analyses	Limited or not tested	Constituent-based	[30]
	Comparative cancer cell models	Methoxylation-dependent differences in antiproliferative activity and selectivity	Comparative structure–activity analysis; direct target not established	Not tested	Constituent-based	[31]
Apigenin	HCC cells and xenograft model	PI3K/AKT/mTOR inhibition, apoptosis, and cytoprotective autophagy	ATG5 silencing and pharmacological autophagy inhibition	Enhanced efficacy following autophagy inhibition	Constituent-based	[32]
	Doxorubicin-resistant HCC cells and xenograft model	Suppression of antioxidant defense and protective autophagy; restoration of drug sensitivity	PI3K/AKT/Nrf2- and miRNA–autophagy pathway interventions	Improved combination efficacy	Constituent-based	[33,34,35]
	Cancer cell models treated with TRAIL or conventional anticancer agents	Enhanced apoptosis and treatment sensitivity	Death-receptor- and mitochondrial-apoptosis-associated analyses	Model dependent	Constituent-based	[36,37]
	Cervical cancer cells	Growth suppression, cell-cycle arrest, and apoptosis	Pathway-associated analyses; direct target not established	Not tested	Constituent-based	[38]
	Tumor cell and angiogenesis-related models	Reduced HIF-1α and VEGF expression through PI3K/AKT/p70S6K1- and HDM2/p53-associated pathways	Pathway-associated analyses	Limited or not tested	Constituent-based	[39]
	Laryngeal carcinoma cells and tumor model receiving radiotherapy	GLUT1 and PI3K/AKT inhibition and enhanced radiosensitivity	Metabolic and pathway-associated analyses	Enhanced radiotherapy response	Constituent-based	[40]
Luteolin	Multiple malignant cell models	DR5-dependent apoptosis	DR5 siRNA and soluble DR5/Fc receptor blockade	Not tested	Constituent-based	[41]
	Metastatic cancer cell models	Reduced migration and invasion through AhR–CXCR4–MMP-associated regulation	Pathway-associated and functional migration/invasion assays	Model dependent	Constituent-based	[42]
	Clear-cell renal cell carcinoma cells and xenograft model	Iron accumulation, glutathione depletion, lipid peroxidation, and HO-1-associated ferroptosis	Ferrostatin-1 and iron-chelator rescue experiments	Reduced xenograft growth	Constituent-based	[43]
	Patient-derived glioma stem cells, macrophage co-cultures, and intracranial model	Reduced IL-6/STAT3 and TGF-β1 signaling and M2-like macrophage polarization	Functional co-culture and tumor microenvironment analyses	Reduced intracranial tumor progression	Constituent-based	[44]
	NCI-H460 lung carcinoma cells	ER-stress-mediated apoptosis and noncanonical autophagy	ER-stress, apoptosis, and autophagy analyses	Not tested	Constituent-based	[45]
	Non-small-cell lung cancer cells receiving radiation	Enhanced radiation-induced apoptosis through the p38/ROS/caspase pathway	ROS and pathway intervention analyses	Not tested	Constituent-based	[46]
	HCT116 colorectal cancer cells	p53-dependent apoptosis and autophagy	p53-associated pathway analyses	Not tested	Constituent-based	[47]
	Laryngeal cancer models receiving radiotherapy	Reduced integrin β1-associated angiogenic signaling and enhanced radiosensitivity	Integrin β1-associated functional analyses	Enhanced radiotherapy response	Constituent-based	[48]
	Prostate cancer cells and tumor model	Suppressed VEGFR2-mediated angiogenesis and tumor growth	Angiogenesis and VEGFR2 pathway analyses	Reduced tumor growth	Constituent-based	[49]
*C. japonicum* extract/acacetin	Rat pharmacokinetic study following oral administration of the extract	Acacetin and six other flavonoids detected in plasma	LC–MS/MS exposure analysis	Systemic exposure demonstrated; no tumor model	Direct plant-specific pharmacokinetic evidence	[50]
Acacetin	Primary CLL cells, normal B cells, isolated mitochondria, and xenograft model	Selective mitochondrial ROS generation and apoptosis in malignant cells	Tumor–normal primary cell and mitochondrial comparisons	Reduced xenograft growth	Constituent-based	[51]
	Gastric cancer cells and xenograft model	EGFR engagement, suppression of STAT3/ERK signaling, and growth inhibition	DARTS, CETSA, and EGFR agonist reversal	Reduced xenograft growth	Constituent-based	[52]
	NSCLC cells and xenograft model	p53–miR-34a-associated growth suppression and PD-L1 reduction	p53 knockdown and miR-34a inhibition	Reduced xenograft growth	Constituent-based	[53]
	Head-and-neck squamous cell carcinoma cells	Mitochondrial and caspase-dependent apoptosis involving the muscarinic M3 receptor	M3-receptor knockdown and apoptosis analyses	Not tested	Constituent-based	[54]
	Oral squamous cell carcinoma cells	Apoptosis associated with MAPK and mitochondrial signaling	Pharmacological and pathway-associated analyses	Not tested	Constituent-based	[55]
Linarin	Glioma cells and xenograft model	Reduced NF-κB/p65 signaling and increased p53- and apoptosis-associated responses	Primarily pathway-associated analyses	Reduced xenograft growth	Constituent-based	[56]
	Colorectal cancer cells and xenograft model	Suppression of HIF-1α/PD-L1 signaling, invasion, and tumor growth	HIF-1α and PD-L1 overexpression rescue	Reduced xenograft growth	Constituent-based	[57]
	Cancer cell models	Cyclin A2-associated cell-cycle arrest and senescence-like responses	Cell-cycle and senescence-associated analyses; genetic rescue not performed	Not tested	Constituent-based	[58]
	MDA-MB-231 triple-negative breast cancer cells, including three-dimensional cultures	Reduced survival and migration; decreased NF-κB p65 and MMP-9 and increased Bax and caspase-3/7	Expression, viability, and migration analyses	Not tested	Constituent-based	[59]

Constituent-based evidence includes studies using isolated or purified compounds that were commercially obtained or derived from botanical sources other than *C. japonicum*; this classification reflects the absence of direct *C. japonicum*-specific evidence and does not imply exclusively commercial sourcing. Abbreviations not defined in the table are listed in the Abbreviations section.

**Table 2:** Experimentally supported molecular targets and pathway dependencies.

Compound	Proposed Target/Critical Mediator	Validation Approach	Principal Downstream Effects	In Vivo Support	Evidence Strength	Major Caveat	Key Reference
Acacetin	EGFR	DARTS; CETSA; EGFR agonist reversal	Reduced EGFR phosphorylation; STAT3/ERK inhibition; apoptosis	Xenograft	Strong	Binding site, affinity and kinase selectivity remain undefined	[52]
Pectolinarigenin	RRM2	RRM2 overexpression rescue; autophagic flux analysis	RRM2 and CDK1 degradation; G2/M arrest	Subcutaneous and orthotopic models	Strong	Direct compound–RRM2 binding not established	[20]
Pectolinarigenin	TOP2A	Target prediction and functional DNA damage analyses	DNA damage; p53-associated G2/M arrest; gemcitabine sensitization	Xenograft	Moderate–strong	Definitive TOP2A cleavage complex trapping requires biochemical confirmation	[21]
Pectolinarigenin	PTEN–PI3K/AKT	PTEN inhibitor and AKT activator experiments	Reduced proliferation and EMT-associated phenotype	No dedicated metastasis model	Moderate	Direct target and full genetic rescue absent	[22]
Pectolinarigenin	STAT3	Phosphorylation and metastasis analyses	Reduced MMP-2/9; increased TIMP2; reduced lung metastasis	Immunocompetent metastasis model	Moderate	Constitutively active STAT3 rescue not performed	[19]
Luteolin	DR5	DR5 siRNA; DR5/Fc receptor blockade	Caspase-8/10/9/3 activation; apoptosis	No	Moderate	Direct ligand–receptor binding remains to be established.	[41]
Apigenin	DR5/TRAIL pathway	DR5/Fc blockade; caspase inhibitors	TRAIL sensitization; caspase activation	No	Moderate	Requires in vivo TRAIL combination validation	[36]
Acacetin	Muscarinic M3 receptor	M3-receptor siRNA	Ca^2+^ and ROS increase; mitochondrial apoptosis	No	Moderate	Receptor dependence does not prove direct binding	[62]
Linarin	HIF-1α/PD-L1	HIF-1α and PD-L1 overexpression rescue; ubiquitination analysis	Reduced growth, invasion and checkpoint-associated signaling	Xenograft	Strong	Immune-mediated PD-L1 function not tested in an intact immune system	[57]
Acacetin	p53–miR-34a	p53 knockdown; miR-34a gain/loss of function	Cell-cycle arrest; apoptosis; PD-L1 reduction	Xenograft	Strong	Clinical relevance of achieved exposure remains unclear	[53]
Apigenin	ATG5-dependent protective autophagy	ATG5 silencing; 3-MA	Increased apoptosis after autophagy inhibition	Xenograft combination	Strong	3-MA lacks complete specificity	[32]
Apigenin	miR-520b–ATG7	miRNA mimic; ATG7-related validation	Reduced protective autophagy; doxorubicin sensitization	Xenograft	Strong	Same resistant-HCC research programme; limited independent replication	[35]
Luteolin	HO-1-associated ferroptosis	Ferrostatin-1; iron chelation; HO-1-pathway inhibition	Labile iron and lipid peroxidation increase	Xenograft	Strong	Direct interaction with HO-1 not demonstrated	[43]
Luteolin	AhR	AhR pharmacological inhibition	CXCR4 and MMP-2/9 reduction; reduced metastasis	Experimental lung metastasis model	Moderate–strong	In vitro and in vivo cancer types differed	[42]
Luteolin	Integrin β1	Overexpression/silencing	VEGFA reduction; antiangiogenesis; radiosensitization	Xenograft	Strong	Direct compound–integrin binding not established	[48]
Cirsimaritin	VEGF/AKT/ERK-associated signaling	Protein expression and phosphorylation analyses	Reduced endothelial tube formation	No	Emerging	Correlative; general endothelial cytotoxicity may contribute	[16]

**Table 3:** Hallmarks of Cancer affected by *C. japonicum*-associated constituents.

Cancer Hallmark/ Enabling Feature	Principal Compounds/ Materials	Representative Molecular Axes	Best-Supported Phenotypes	Overall Evidence	Principal Limitation	Key Reference(s)
Sustaining proliferative signaling	Pectolinarigenin; acacetin; linarin; apigenin	EGFR; RRM2–CDK1; TOP2A; PI3K/AKT/mTOR; cyclins/CDKs	Reduced colony formation; G1 or G2/M arrest; xenograft suppression	Strong	Plant-specific mechanistic evidence is sparse	[20,21,23,32,52,53,56,58]
Evading growth suppressors	Acacetin; linarin; luteolin	p53–miR-34a; p21; RB; cyclin A2/CHEK1	p53-associated arrest; senescence; reduced RB phosphorylation	Moderate	Durability of arrest and post-washout recovery rarely assessed	[47,53,56,58]
Resisting cell death	Pectolinarigenin; luteolin; apigenin; acacetin; linarin	DR5; M3 receptor; Bax/Bcl-2; caspases; mitochondrial ROS	Intrinsic/extrinsic apoptosis; tumor-selective apoptosis	Strong	Primary initiating targets are often unknown	[18,36,41,51,54,56]
Autophagy adaptation	Apigenin; pectolinarigenin; luteolin	ATG5; ATG7; RRM2 degradation; late-flux blockade	Protective, death-promoting or cargo-selective autophagy	Strong	LC3-II is often interpreted without complete flux analysis	[20,21,32,35,45]
Ferroptosis/redox vulnerability	Luteolin; *CJ-AuNPs*	HO-1; Fe^2+^; GPX4; GSH; lipid peroxidation	Ferroptosis rescue by inhibitors; xenograft inhibition	Moderate–strong for luteolin; emerging for plant-derived formulation	Conventional plant extract has not been validated as a ferroptosis inducer	[17,43]
Tumor-promoting inflammation	Linarin; pectolinarigenin; luteolin	NF-κB; IL-6/STAT3; TGF-β1/SMAD3	Reduced inflammatory signaling; altered macrophage phenotype	Moderate	Most studies measure markers rather than immune dependency	[19,44,56]
Activating invasion and metastasis	Pectolinarigenin; luteolin; apigenin; linarin	STAT3–MMP; PTEN–AKT; AhR–CXCR4; integrin β1–FAK; HIF-1α	Reduced migration/invasion; fewer lung metastases	Moderate–strong	Many studies use migration assays rather than spontaneous metastasis models	[19,22,38,42,48,57]
Inducing/accessing vasculature	Cirsimaritin; luteolin; apigenin	VEGF/VEGFR2; Notch1; HIF-1α; integrin β1	Tube formation, vascular sprouting and microvessel density reduction	Moderate–strong	Direct plant evidence is limited to endothelial surrogate assays	[16,39,48,49]
Avoiding immune destruction	Pectolinarigenin; linarin; luteolin	CD8 recruitment; HIF-1α/PD-L1; IL-6/STAT3–TAM signaling	Increased CD8^+^ cells; reduced PD-L1; altered macrophage polarization	Moderate	Immune cell depletion and checkpoint combination studies are rare	[19,44,57]
Deregulating cellular energetics	Apigenin; acacetin; luteolin	GLUT1; mitochondrial ROS; Nrf2; HO-1/iron	Radiosensitization; selective mitochondrial injury; ferroptosis	Moderate	Metabolic flux and isotope-tracing studies are largely absent	[33,40,43,51]
Senescence/phenotypic plasticity	Linarin; luteolin	Cyclin A2/CHEK1; IL-6/STAT3; TGF-β1	Senescence-like arrest; altered glioma stem cell/TAM crosstalk	Emerging	Persistence, SASP and relapse potential not assessed	[44,58]
Therapy resistance	Apigenin; luteolin; pectolinarigenin	Nrf2; ATG7; GLUT1; integrin β1; TOP2A/autophagy	Doxorubicin, gemcitabine and radiation sensitization	Moderate–strong	Formal synergy and normal-tissue toxicity analyses remain incomplete	[21,33,35,40,46,48]

**Table 4:** Evidence strength grading of principal anticancer mechanisms.

Mechanism	Cellular Evidence	Functional Rescue/ Inhibition	Animal Evidence	Direct-Target Evidence	Plant-Specific Evidence	Overall Grade	Key Reference
EGFR-associated growth signaling	Strong	EGFR agonist reversal	Yes	DARTS/CETSA	No	Strong	[52]
RRM2–CDK1 cell-cycle control	Strong	RRM2 overexpression rescue	Yes, including orthotopic model	Mediator dependency established; direct binding absent	No	Strong	[20]
TOP2A-associated DNA damage	Strong	Autophagy and drug combination analyses	Yes	Incomplete biochemical confirmation	No	Moderate–strong	[21]
PI3K/AKT/mTOR inhibition	Extensive	Occasional inhibitor/activator studies	Multiple	Generally absent	Limited	Moderate	[22,23,32]
DR5-mediated apoptosis	Strong	DR5 siRNA and receptor blockade	Limited	Receptor dependency, not direct ligand binding	No	Moderate	[36,41]
Mitochondrial apoptosis	Extensive	Caspase and MAPK inhibitors in selected studies	Multiple	Primary mitochondrial target unknown	Limited	Moderate–strong	[18,51,55]
Protective autophagy	Strong	ATG5 silencing, 3-MA and miRNA intervention	Yes	Functional pathway evidence	No	Strong	[32,35]
Autophagic degradation of oncogenic proteins	Strong	RRM2 rescue	Yes	Cargo/mediator validation	No	Strong	[20]
Ferroptosis	Strong for luteolin; moderate for *CJ-AuNPs*	Ferrostatin-1 and iron chelator rescue	Yes	Direct molecular target incomplete	Formulation-specific	Strong for luteolin; moderate for *CJ-AuNPs*	[17,43]
STAT3-associated metastasis/inflammation	Strong cellular association	Limited direct rescue	Metastasis model	Direct binding absent	No	Moderate	[19,44]
HIF-1α/PD-L1 regulation	Strong	Dual overexpression rescue	Xenograft	Direct binding absent	No	Strong	[57]
Antiangiogenesis	Strong constituent literature	Notch1/integrin β1 rescue in selected studies	Multiple	Mostly indirect	One limited extract study	Moderate–strong (constituent-based); Emerging (direct plant-specific)	[16,48,49]
Immune microenvironment remodelling	Moderate	Limited immune cell dependency tests	Selected immunocompetent/intracranial models	Not applicable	Early immune index evidence	Moderate	[14,19,44]
Chemosensitization	Strong in selected models	miRNA/autophagy pathway interventions	Yes	Context-specific	No	Strong	[21,33,35]
Radiosensitization	Strong clonogenic and xenograft evidence	p38/ROS or integrin β1 interventions	Yes	Direct target incomplete	No	Strong	[40,46,48]
Senescence induction	Initial evidence	No genetic rescue	No	No	No	Emerging	[58]

Evidence strength was graded using four predefined categories. Strong evidence required functional dependency demonstrated by genetic or well-controlled pharmacological intervention together with relevant in vivo validation; claims of direct molecular targeting additionally required target engagement evidence. Moderate–strong evidence comprised consistent cellular findings supported by in vivo evidence and either functional intervention or target engagement analysis, but lacking complete dependency, direct-target, or independent validation. Moderate evidence comprised coherent cellular findings accompanied by either limited functional intervention or in vivo support, but not both. Emerging evidence was based mainly on associative, single-model, or preliminary findings without adequate functional intervention or relevant in vivo validation. Evidence was classified as not established when meaningful experimental support for a particular mechanism or hallmark was unavailable. Plant specificity was assessed separately and did not increase the mechanistic evidence grade.

**Table 5:** Methodological and translational limitations of the current evidence.

Issue	Current Status	Consequence for Interpretation	Recommended Experimental Strategy	Priority	Key Reference
Plant versus constituent evidence	Most advanced mechanisms derive from purified compounds obtained commercially or from other plants	Purified compound activity may be incorrectly attributed to *C. japonicum* preparations	Standardized extract versus purified compound comparison; constituent depletion and reconstitution studies	Very high	[14,20,52]
Extract standardization	Variable extraction methods and incomplete quantitative profiles	Poor reproducibility and uncertain active dose	Validated LC–MS/HPLC marker panels; batch-to-batch acceptance ranges	Very high	[15,16]
Direct-target validation	Most mechanisms rely on phosphorylation or expression changes	Primary targets cannot be separated from downstream stress responses	CETSA, DARTS, thermal proteome profiling, affinity chemoproteomics and CRISPR screens	Very high	[20,52]
Reliance on molecular docking	Commonly used as evidence of direct targeting	High risk of overclaiming binding and specificity	Biochemical binding, competition, kinetic and mutant-rescue assays	High	[58,78]
Pharmacokinetics and bioavailability	Rarely measured	In vitro active concentrations may be clinically unattainable	Plasma/tissue PK, metabolite profiling, protein binding and tumor exposure	Very high	[50]
Glycoside–aglycone conversion	Pectolinarin and pectolinarigenin often discussed together	Absorption and active molecular species remain uncertain	Gut microbiome metabolism, hydrolysis and comparative PK studies	High	[50]
Model relevance	Heavy reliance on 2D immortalized cell lines	Tumor heterogeneity and microenvironment are poorly represented	Organoids, spheroids, PDX and orthotopic models	High	[59]
Immune evaluation	Many in vivo studies use immunodeficient xenografts	PD-L1 and immune effects cannot be interpreted functionally	Syngeneic or humanized models; immune cell depletion; checkpoint combinations	Very high	[19,57]
Metabolic characterization	Mostly ROS, GLUT1 or membrane potential measurements	Claims of metabolic reprogramming remain incomplete	Metabolomics, isotope tracing, Seahorse analysis and lipidomics	High	[46,51]
Autophagy assessment	LC3-II frequently used without full flux analysis	Autophagy induction may be confused with degradation blockade	Tandem LC3 reporter, lysosomal blockade, p62 turnover and ATG manipulation	High	[21,32]
Combination studies	Often lack formal synergy calculations	Additive toxicity may be mislabelled as synergism	Combination index analysis, exposure sequencing and normal-cell testing	High	[21,33]
Normal-cell selectivity	Infrequently evaluated	Therapeutic window remains unknown	Matched normal cells, organoids and normal-tissue toxicology	Very high	[41,51]
Independent replication	Several mechanisms originate from a single research group or model	Generalizability remains uncertain	Multi-centre validation and independent model replication	High	[33,34,35]

**Table 6:** Translational positioning and priority development needs of *C. japonicum*-associated materials.

Compound/ Material	Distinctive Translational Value	Principal Unresolved Limitation	Developmental Position	Priority Next Step
Pectolinarigenin	Broad target-oriented evidence across multiple cancer models, including functional validation of selected molecular dependencies [19,20,21]	Direct targets remain incompletely defined, and systemic and tumor exposure data are insufficient	Advanced preclinical constituent candidate	Confirm direct target engagement and establish quantitative exposure–response relationships
Acacetin	Functional evidence involving EGFR and p53–miR-34a signaling, together with preliminary tumor-selectivity evidence [51,52,53]	Pharmacokinetics of purified acacetin, tumor exposure, and target selectivity remain poorly characterized	Target-oriented preclinical candidate	Determine pharmacokinetics, tumor-selective target engagement, and therapeutic window
Luteolin	Broad activity involving regulated cell death, ferroptosis, angiogenesis, metastasis, and tumor–microenvironment regulation [41,43,46]	Evidence is weakly specific to *C. japonicum*, and systemic exposure is generally limited or insufficiently characterized	Broad-mechanism preclinical candidate	Improve formulation and exposure and identify mechanism-linked predictive biomarkers
Apigenin	Extensive evidence for context-dependent autophagy regulation and sensitization to anticancer treatments [32,33,36]	The functional role of autophagy varies among tumor models, and clinically achievable exposure remains uncertain	Combination-oriented preclinical candidate	Define tumor contexts in which autophagy modulation improves treatment response and verify achievable exposure
Linarin	Emerging evidence involving inflammatory signaling, HIF-1α/PD-L1 regulation, and tumor growth suppression [56,57]	Findings are recent, incompletely replicated, and supported by limited pharmacokinetic information	Early mechanistic candidate	Independently replicate the findings in immunocompetent and clinically relevant models
Cirsimaritin	Direct association with a *C. japonicum* var. *maackii* preparation and preliminary antiangiogenic and cytotoxic activity [16,29]	Direct targets, in vivo vascular effects, exposure, and the contribution of the individual constituent remain unresolved	Early preclinical candidate	Establish direct targets and validate antiangiogenic efficacy in vivo
Pectolinarin	Plant-associated glycoside with preliminary cytotoxic and stress-response evidence [26,27]	The active molecular species, glycoside–aglycone conversion, pharmacokinetics, and anticancer efficacy remain uncertain	Discovery-stage constituent	Compare metabolism, exposure, target engagement, and efficacy directly with pectolinarigenin
Standardized *C. japonicum* extract	Directly relevant botanical material with preliminary antitumor and antiangiogenic evidence [14,15,16]	Validated standardization, quantitative marker profiles, pharmacokinetics, and mechanism-linked efficacy are lacking	Botanical discovery stage	Develop a chemically standardized preparation and establish batch-specific exposure and efficacy criteria
*CJ-AuNPs*	Formulation-specific ferroptosis-associated activity with preliminary in vivo efficacy [17]	The relative contributions of the gold core and botanical coating and the biodistribution of the nanoparticles remain unclear	Early formulation candidate	Separate nanoparticle–core and phytochemical contributions and characterize biodistribution and safety

Developmental position is a qualitative translational designation and is distinct from the mechanistic evidence strength categories defined above.

**Table 7:** Compound–hallmark evidence matrix for *C. japonicum*-associated materials.

Compound/ Material	Proliferation/Growth Suppression	Cell Death	Autophagy	Ferroptosis/Redox	Inflammation/TME	Invasion/Metastasis	Angiogenesis/Hypoxia	Immune Evasion	Therapy Resistance	Key Reference(s)
Pectolinarigenin	S	MS	S	E	M	M	E	E	MS	[18,19,20,21,22,23]
Apigenin	M	MS	S	M	E	M	M	E	S	[32,33,34,35,36,37,38,39,40]
Luteolin	M	MS	M	S	MS	MS	S	M	S	[41,42,43,44,45,46,47,48,49]
Acacetin	S	MS	—	MS	E	E	E	MS	E	[51,52,53,54,55]
Linarin	S	M	—	—	M	S	M	MS	E	[56,57,58,59]
Cirsimaritin	E	E	—	E	E	E	E	—	—	[16,29,30]
Pectolinarin	E	E	E	—	—	—	—	—	—	[26,27]
Phenylpropanoid glycosides	E	E	—	—	—	—	—	—	—	[13]
*C. japonicum* flavone preparation and isolated flavones	M	—	—	—	E	—	—	E	—	[14,15]
*C. japonicum* var. *maackii* extract/cirsimaritin	E	E	—	—	E	E	E	—	—	[16]
*CJ-AuNPs*	M	M	—	M	—	—	—	—	—	[17]

Evidence grading: S, strong; MS, moderate–strong; M, moderate; E, emerging; —, not established. Strong evidence required functional dependency demonstrated by genetic or well-controlled pharmacological intervention together with relevant in vivo validation; direct-target claims additionally required target engagement evidence. Moderate–strong evidence comprised consistent cellular and in vivo findings supported by either functional intervention or target engagement analysis but lacking complete dependency, direct-target, or independent validation. Moderate evidence comprised coherent cellular findings accompanied by either limited functional intervention or in vivo support, but not both. Emerging evidence was based mainly on preliminary, associative, or single-model findings without adequate functional intervention and relevant in vivo validation. Ratings indicate evidence strength rather than effect magnitude, clinical efficacy, or botanical specificity.

## Data Availability

No new data were created or analyzed in this study. Data sharing is not applicable to this article.

## Abbreviations

The following abbreviations are used in this manuscript:

3-MA	3-Methyladenine
AhR	Aryl hydrocarbon receptor
AKT	Protein kinase B
ATF6	Activating transcription factor 6
ATG	Autophagy-related gene/protein
CDK1	Cyclin-dependent kinase 1
CETSA	Cellular thermal shift assay
CJ-AuNPs	*Cirsium japonicum var. maackii*-mediated gold nanoparticles
CLL	Chronic lymphocytic leukemia
CRC	Colorectal cancer
CXCR4	C-X-C motif chemokine receptor 4
DARTS	Drug affinity responsive target stability
DR5	Death receptor 5
EGFR	Epidermal growth factor receptor
eIF2α	Eukaryotic translation initiation factor 2 alpha
EMT	Epithelial–mesenchymal transition
ER	Endoplasmic reticulum
ERK	Extracellular signal-regulated kinase
GLUT1	Glucose transporter 1
GPX4	Glutathione peroxidase 4
HCC	Hepatocellular carcinoma
HIF-1α	Hypoxia-inducible factor 1 alpha
HO-1	Heme oxygenase 1
HUVEC	Human umbilical vein endothelial cell
IL-6	Interleukin 6
IRE1	Inositol-requiring enzyme 1
MAPK	Mitogen-activated protein kinase
MMP	Matrix metalloproteinase
mTOR	Mechanistic target of rapamycin
NF-κB	Nuclear factor kappa B
NSCLC	Non-small-cell lung cancer
Nrf2	Nuclear factor erythroid 2-related factor 2
PD-L1	Programmed death-ligand 1
PERK	Protein kinase RNA-like endoplasmic reticulum kinase
PI3K	Phosphoinositide 3-kinase
PK	Pharmacokinetics
PTEN	Phosphatase and tensin homolog
ROS	Reactive oxygen species
RRM2	Ribonucleotide reductase regulatory subunit M2
SASP	Senescence-associated secretory phenotype
STAT3	Signal transducer and activator of transcription 3
TGF-β	Transforming growth factor beta
TOP2A	DNA topoisomerase II alpha
TRAIL	Tumor necrosis factor-related apoptosis-inducing ligand
VEGF	Vascular endothelial growth factor
VEGFR2	Vascular endothelial growth factor receptor 2
